# Breaking the Rhythm: Extracorporeal Membrane Oxygenation (ECMO) as a Life Saver in a Case of Resistant Arrhythmia and Cardiac Arrest

**DOI:** 10.7759/cureus.99196

**Published:** 2025-12-14

**Authors:** Alaa B Fadl, Aya Fadl, Rashid Nadeem

**Affiliations:** 1 Intensive Care Medicine, Dubai Hospital, Dubai, ARE; 2 General Surgery, Dubai Hospital, Dubai, ARE

**Keywords:** arrhythmia-induced cardiomyopathy, cardiac arrest, ecmo outcome, extra-corporeal membrane oxygenation, implantable cardioverter defibrillator, refractory ventricular arrhythmia

## Abstract

Refractory ventricular arrhythmias and cardiac arrest in younger patients are rare, life-threatening events, particularly when standard resuscitation fails. Extracorporeal membrane oxygenation (ECMO) provides temporary circulatory support, enabling hemodynamic stabilization, myocardial recovery, and time for diagnostic and therapeutic interventions.

We report the case of a 40-year-old East African woman, medically free at baseline, who developed refractory ventricular fibrillation (VF) and cardiogenic shock during elective myomectomy on 12^th^ August 2024. She underwent 45 minutes of cardiopulmonary resuscitation (CPR) before the return of spontaneous circulation. Echocardiography revealed severe left ventricular (LV) dysfunction with an ejection fraction (EF) of approximately 10%. Veno-arterial ECMO (VA-ECMO) was initiated 15 hours after the first VF episode on 13^th^ August 2024 per institutional protocols; support continued for six days until 19^th^ August 2024. During ECMO, coronary assessment, continuous rhythm monitoring, and serial echocardiography were performed to guide management and systematically exclude reversible causes, including ischemia, myocarditis, embolic events, and electrolyte disturbances. An intra-aortic balloon pump (IABP) was added on 14^th^ August 2024 and removed on 20^th^ August 2024.

Post-event discussions revealed a previously unrecognized strong family history of sudden cardiac death. Despite maximal antiarrhythmic therapy, recurrent VF and nonsustained ventricular tachycardia (VT) persisted. A temporary pacemaker was inserted on 30^th^ August 2024, and an implantable cardioverter-defibrillator (ICD) was placed for secondary prevention, risk stratification, and management of persistent diagnostic uncertainty. Genetic testing was performed due to the newly discovered family history, including a long QT gene panel (ordered 5^th^ September 2024, reported 31^st^ October 2024) and proband whole exome sequencing (WES; ordered 5^th^ September 2024, reported 27^th^ February 2025). WES identified a heterozygous likely pathogenic variant in the AIP gene and two variants of uncertain significance in TTN, while the long QT panel was negative; none fully explained the arrhythmia. Familial screening and clinical correlation were recommended.

The patient achieved full recovery, with normalization of cardiac function and improved strain parameters by hospital discharge. This case highlights VA-ECMO as a bridge to myocardial recovery in refractory arrhythmias, underscores systematic exclusion of reversible triggers, and demonstrates that combined ECMO, IABP, and ICD therapy can be life-saving in preventing recurrent cardiac arrest and sudden cardiac death.

## Introduction

Refractory ventricular arrhythmias and cardiac arrest in younger patients are rare but life-threatening events, particularly when standard resuscitation fails. Extracorporeal membrane oxygenation (ECMO) provides temporary circulatory support, enabling hemodynamic stabilization, myocardial recovery, and time for diagnostic and therapeutic interventions [1]. While perioperative cardiac arrests are uncommon, regional data indicate that arrhythmic causes constitute a meaningful proportion of these events, supporting consideration of advanced resuscitative measures such as ECMO in surgical populations [2,3]. Estimates of in-hospital cardiac arrest in the United States reach approximately 292,000 adults annually, and a tertiary care hospital study in the United Arab Emirates reported an incidence of 11.7 per 1,000 admissions [1,2]. However, data specific to refractory ventricular arrhythmias or ECMO use remain limited, particularly in perioperative settings [4].

Veno-arterial ECMO (VA ECMO) was selected over isolated intra-aortic balloon pump (IABP) support or prolonged cardiopulmonary resuscitation (CPR) due to sustained hemodynamic instability and refractory arrhythmias. ECMO provided systemic organ perfusion while allowing administration of antiarrhythmic therapies that could exacerbate hypotension, offering a bridge to myocardial recovery when conventional measures were insufficient [5].

The patient exhibited transient severe left ventricular (LV) dysfunction, generating diagnostic uncertainty and a broad differential diagnosis, which includes the following: 1. arrhythmia-induced cardiomyopathy: severe arrhythmias can cause acute LV dysfunction, which may recover rapidly once rhythm control is achieved. 2. Stress (takotsubo) cardiomyopathy: transient regional wall motion abnormalities, often triggered by perioperative or emotional stress. 3. Dilated cardiomyopathy: usually presents with chronic LV dilation and systolic dysfunction. 3. Hypertrophic cardiomyopathy: characterized by asymmetric septal hypertrophy and diastolic dysfunction. 4. Arrhythmogenic cardiomyopathy: fibrofatty infiltration of the right or left ventricle, predisposing to ventricular arrhythmias.

The subtype could not be definitively determined initially due to the absence of chronic remodeling, rapid normalization of ventricular function, and overlapping clinical features.

A structured diagnostic evaluation was performed during the period of hemodynamic support, including cardiac imaging, rhythm monitoring, and sequential assessments to exclude reversible and underlying inherited causes [1,4,5].

This case emphasizes the importance of timely recognition of perioperative triggers, systematic evaluation of reversible causes, the life-saving role of ECMO in refractory arrhythmias, and integration of advanced diagnostic strategies to guide management, risk stratification, and preventive care.

## Case presentation

A 40-year-old female patient, mother of three children (last delivery in 2015), with no history of diabetes or hypertension, was transferred for management of refractory ventricular arrhythmias. A previously unknown strong family history of sudden cardiac death was revealed, as both her brother and sister had died at age 27. She had a history of multiple uterine fibroids and prior right salpingectomy and right oophorectomy in June 2024 for a ruptured ectopic pregnancy, with an uneventful postoperative course.

**Figure 1:**
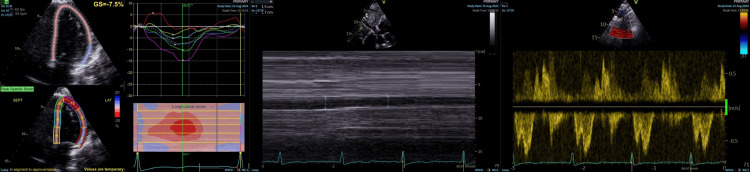
Speckle-tracking echocardiography and Doppler assessment of LV function Severely reduced LV systolic function (EF 25%–30%) with global strain of –8% and regional variability. LVOT VTI 8.5 cm; LV size normal, no pericardial effusion. LV: left ventricular; EF: ejection fraction; LVOT: LV outflow tract; VTI: velocity time integral

On 12^th^ August 2024, she was admitted electively for open myomectomy. Approximately 90 minutes into the procedure, immediately following uterine manipulation, she developed refractory ventricular fibrillation (VF). CPR was initiated immediately and continued for 45 minutes until return of spontaneous circulation, during which she received 15 intraoperative direct current shocks and intravenous amiodarone, lidocaine, magnesium sulfate, and calcium gluconate. Post-resuscitation echocardiography revealed severe global LV dysfunction (LV ejection fraction (EF) 25%-30%), moderate to severe mitral regurgitation, and prominent trabeculations (Figure 1). 

Early laboratory evaluation showed elevated lactate (9.8 mmol/L), mildly elevated cardiac biomarkers (troponin I 0.36 ng/mL, creatine kinase-myocardial band (48 U/L), and borderline electrolytes (potassium: 3.5 mmol/L; magnesium: 1.7 mmol/L). Coronary CT angiography within the first six hours excluded coronary ischemia and embolic events.

Due to persistent VF and cardiogenic shock unresponsive to antiarrhythmics and inotropes, VA-ECMO was initiated one day after surgery, once coagulopathy stabilized. VA-ECMO was chosen for full hemodynamic support while allowing antiarrhythmic therapy to act. She remained sedated with midazolam and fentanyl, mechanically ventilated, and supported with norepinephrine and dobutamine. Cardiac function gradually improved (LVEF 50%-55%), allowing ECMO decannulation. Persistent low cardiac output prompted insertion of an IABP two days post surgery to reduce LV afterload and enhance coronary perfusion.

**Figure 2:**
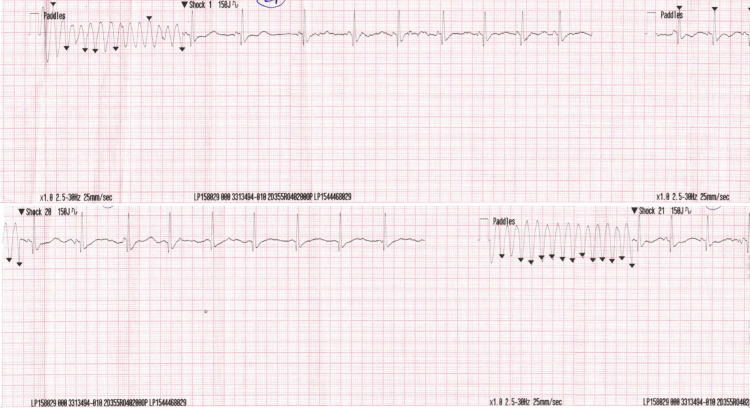
Electrocardiogram rhythm strips Recurrent ventricular tachycardia/ventricular fibrillation episodes requiring more than 20 defibrillation shocks, with only transient return to sinus rhythm.

**Figure 3:**
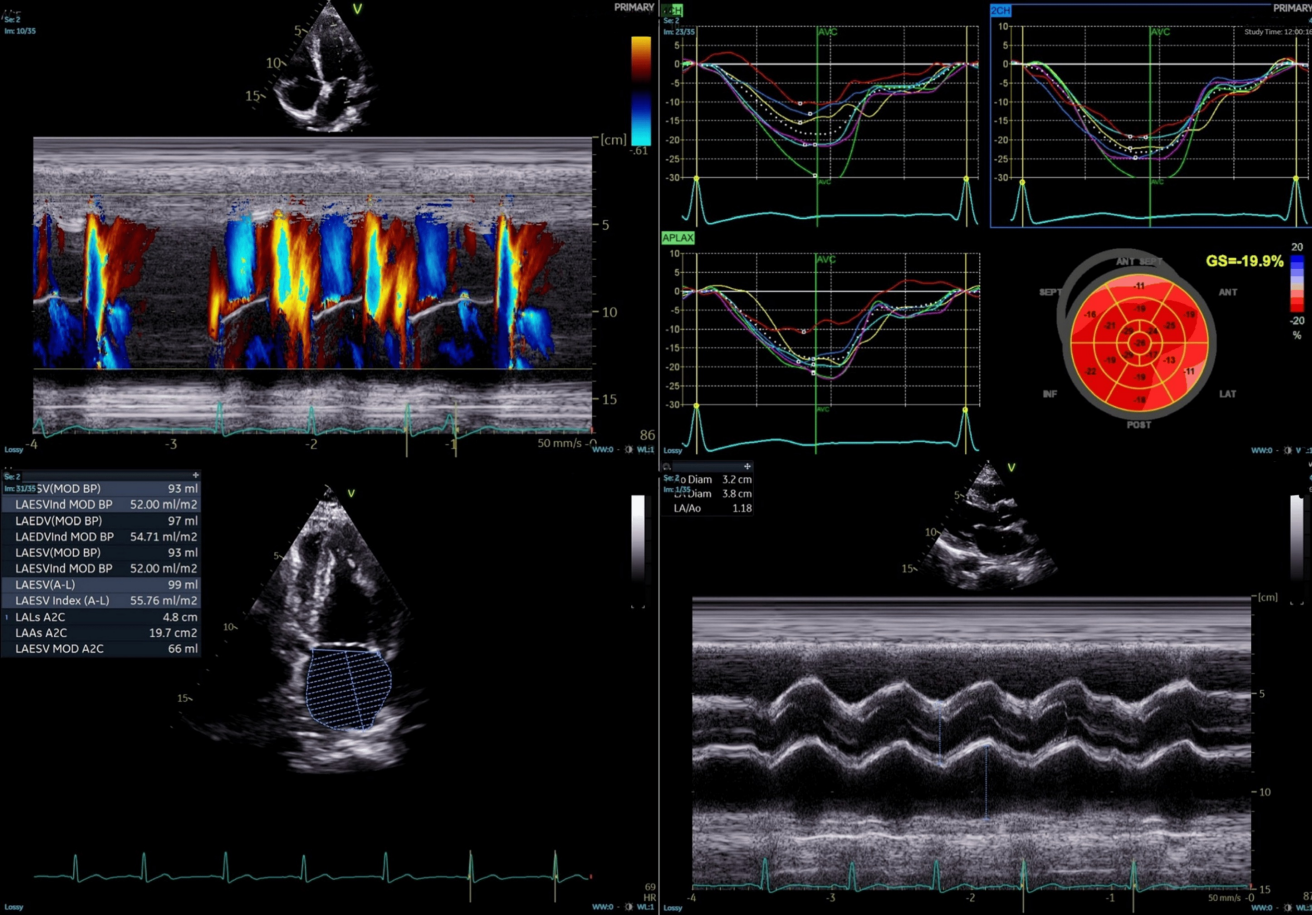
Transthoracic echocardiogram (TTE) TTE after laparotomy and recurrent ventricular arrhythmias shows severe left ventricular dysfunction with an ejection fraction of 25%, likely due to repeated shocks and myocardial stress, consistent with acute systolic dysfunction during an electrical storm.

She subsequently developed abdominal wound dehiscence, which was repaired emergently under spinal anesthesia. Later that evening, recurrent VF and non-sustained ventricular tachycardia (VT) occurred, documented on ECG (Figure 2), with potassium dropping to 3.2 mmol/L and magnesium to 1.6 mmol/L, corrected promptly. Temporary overdrive pacing at 100 beats per minute (bpm) was initiated to suppress pause-dependent arrhythmias and prevent the R-on-T phenomenon. On 29 August 2024, polymorphic VT associated with hypokalemia (2.6 mmol/L) occurred and was treated with IV potassium and magnesium. She experienced a generalized tonic-clonic seizure on lidocaine infusion, managed with sedation and levetiracetam. Amiodarone infusion restarted on 31^st^ August 2024 reduced VT/VF episodes, although echocardiography at this stage showed LVEF 25% (Figure 3).

**Figure 4:**
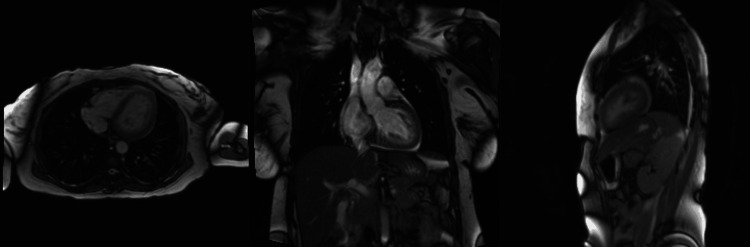
Cardiac magnetic resonance imaging (CMRI) The CMRI shows normal biventricular size and function, with mild septal T1 elevation suggesting subtle inflammation. There is minimal pericardial enhancement consistent with mild chronic pericarditis, and minimal bilateral pleural effusion, with no evidence of cardiomyopathy.

Cardiac magnetic resonance imaging (CMRI), performed after a 72-hour amiodarone washout, demonstrated normal biventricular morphology, mild pericardial enhancement consistent with chronic pericarditis, and no evidence of cardiomyopathy; a viral panel and inflammatory markers excluded myocarditis (Figure 4). Genetic evaluation included a long QT gene panel (38 genes, negative) and whole exome sequencing (WES), which revealed a heterozygous likely pathogenic variant in the AIP gene (c.350del, p.Gly117AlafsTer39) and two variants of uncertain significance in TTN (c.30389A>G, p.Glu10130Gly; c.12494A>G, p.Glu4165Gly). No pathogenic variants fully explained her arrhythmic phenotype. WES was performed using standard clinical capture and next-generation sequencing; whole-genome sequencing was not performed. Familial genetic study and counseling were recommended.

**Figure 5:**
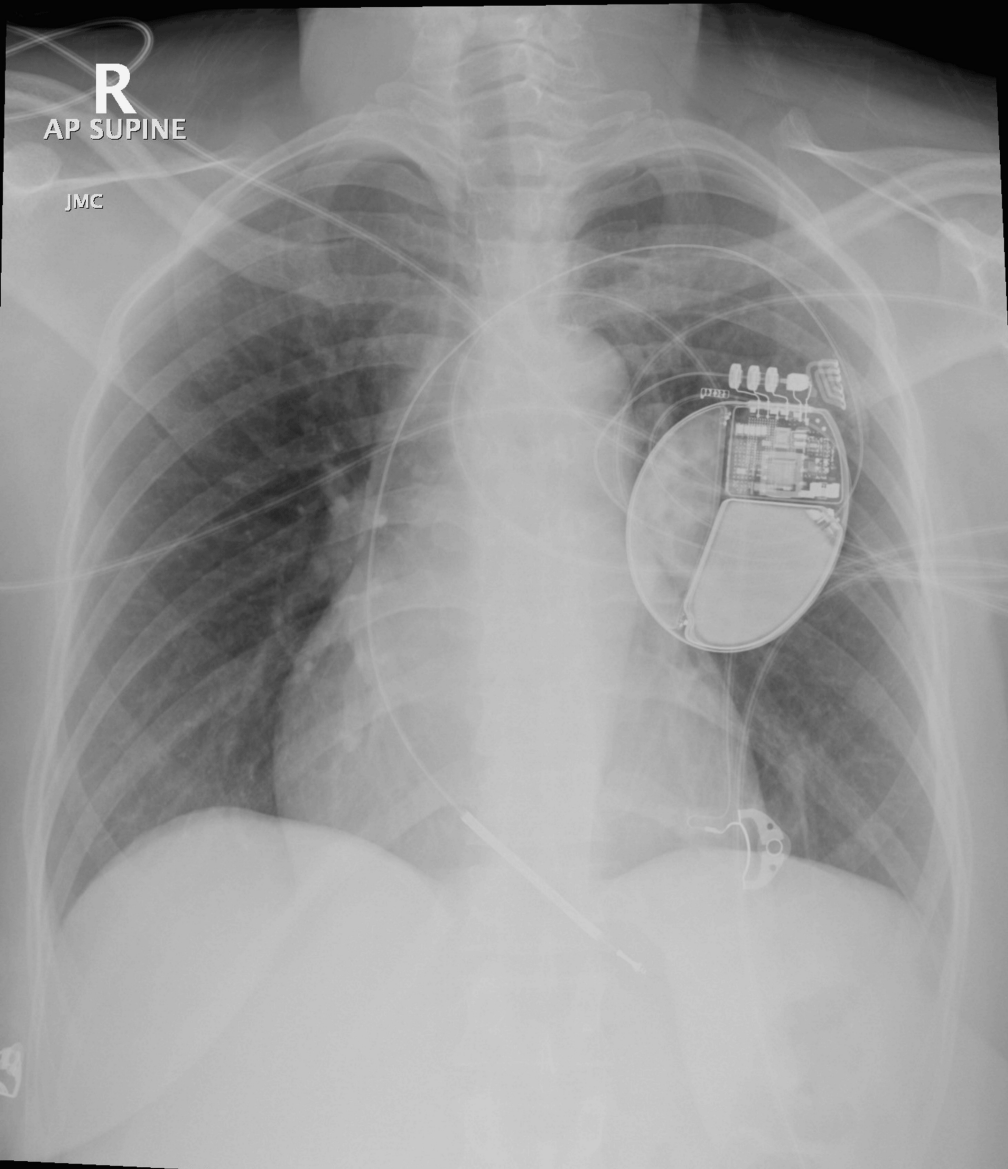
Mobile chest X-ray, anteroposterior (AP) view in supine position AP supine chest X-ray shows a dual-chamber implantable cardioverter-defibrillator (ICD) in the left pectoral area with well-positioned leads. Lungs are clear with no pneumothorax or lead issues. Overall, ICD placement appears satisfactory with no immediate complications.

For secondary prevention of sudden cardiac death, a dual-chamber implantable cardioverter-defibrillator (ICD) was implanted with overdrive pacing, anti-tachycardia pacing (ATP) for VT zone 170-200 bpm, and shock therapy for VF >220 bpm (Figure 5). 

Post-ICD monitoring for seven days revealed no device therapies. She was discharged clinically stable, asymptomatic, with LVEF normalized to 60%, neurologically intact, and without renal dysfunction or ongoing arrhythmias. Amiodarone therapy was intended as temporary, with follow-up for ICD interrogation at one month and echocardiography at three months. The final diagnosis was refractory VF with transient arrhythmia-induced cardiomyopathy, successfully managed with VA-ECMO, IABP, temporary pacing, antiarrhythmic therapy, and ICD implantation.

## Discussion

This clinical case report is unique due to the combination of an unexpected sudden intraoperative cardiac arrest in an otherwise healthy 40-year-old female patient. The situation was further complicated by a refractory arrhythmia requiring advanced resuscitation techniques, including VA-ECMO. Subsequent investigations revealed a previously unknown strong family history of sudden cardiac death, with both her brother and sister dying at age 27. This information was obtained retrospectively during a detailed family interview and genetic counseling session, and although it did not change acute management, it informed risk stratification and follow-up planning.

Cardiac arrest during non-cardiac surgery is rare, occurring in only 0.8-4.3 per 10,000 procedures [6], and survival rates have not shown significant improvement. VF and VT account for nearly 70% of intraoperative cardiac arrests [7]. Some arrhythmias are refractory to standard treatments, necessitating advanced interventions such as catheter ablation, ICDs, or surgery [8]. Electrophysiological studies are commonly used to identify arrhythmia substrates and guide therapy [9]. Structural heart disease often coexists with arrhythmias, particularly in complex congenital heart disease, where atrial arrhythmias are prevalent, and ventricular arrhythmias carry high mortality [10].

Antiarrhythmic therapy is essential for VF management. In our patient, lidocaine infusion (1-2 mg/min, titrated in the ICU) and amiodarone infusion (300 mg IV loading, then 900 mg/24 h) were used for refractory VF. Magnesium sulfate (2 g IV) and calcium gluconate (10 mL of 10% solution IV) were administered repeatedly for suspected electrolyte-related triggers. Long-term therapy included oral amiodarone (200 mg daily), bisoprolol (5 mg daily), and lisinopril (5 mg daily), providing rhythm control, rate control, and neurohormonal blockade. Procainamide, though effective in trials [11], was unavailable in our center, highlighting real-world limitations. Even with treatment, 20%-50% of patients have recurring arrhythmias within five years, often needing device-based therapies [12]. 

VA-ECMO can be used during cardiac arrest as part of extracorporeal CPR, maintaining organ perfusion while the underlying cause is addressed. Average time from CPR initiation to VA-ECMO cannulation is approximately 104 minutes [13]. Short-duration ECMO support (≤4 days) is associated with increased mortality, while optimal outcomes are generally observed when ECMO is weaned around day four [14,15]. In this case, VA-ECMO was initiated one day postoperatively due to intraoperative coagulopathy and logistic considerations, allowing safe femoral cannulation. It provided full circulatory support, enabling antiarrhythmic therapy to take effect. Persistent low cardiac output despite ECMO prompted IABP insertion, which reduced left ventricular afterload and augmented coronary perfusion, facilitating recovery.

The differential diagnosis for transient severe LV dysfunction included arrhythmia-induced cardiomyopathy, stress (takotsubo) cardiomyopathy, and primary dilated cardiomyopathy. Rapid EF recovery on echocardiography, absence of scar on cardiac MRI, and lack of late gadolinium enhancement supported arrhythmia-induced or stress-related cardiomyopathy. Mild chronic pericarditis was noted on MRI, corroborated by negative viral and inflammatory markers, but no myocarditis was detected. MRI was performed after a 72-hour amiodarone washout to avoid confounding enhancement patterns.

Genetic evaluation included a long QT gene panel (38 genes) and WES. WES revealed a heterozygous likely pathogenic variant in the AIP gene and two variants of uncertain significance in TTN, but no definitive pathogenic variants explained her arrhythmia. Whole-genome sequencing was not performed. Negative results reduce the likelihood of a monogenic syndrome but do not fully exclude inherited channelopathies, highlighting the need for continued family surveillance.

The electrophysiological study was deferred due to patient instability and ongoing VA-ECMO/IABP support. ICD implantation was prioritized for secondary prevention, with dual-chamber ICD settings including overdrive pacing, ATP for VT 170-200 bpm, and shock therapy for VF >220 bpm. Post-implant monitoring for seven days revealed no device therapies. A deferred EP study could assess inducibility and substrate characterization during follow-up.

Perioperative triggers likely included surgical stress, catecholamine surge, anesthetic exposure, and electrolyte shifts, with documented hypokalemia (2.6 mmol/L) acting as a key arrhythmogenic trigger. Frequent electrolyte monitoring and prompt replacement were critical to prevent recurrent arrhythmias. Catecholamine exposure from inotropes may have also contributed.

This case is distinctive because it involves a young patient with no prior cardiac disease, transient LV dysfunction, refractory VF, and negative genetic testing. VA-ECMO and IABP served as a lifesaving bridge to recovery, highlighting the importance of advanced mechanical support in perioperative arrhythmic storms. The report contributes novel insight into perioperative triggers, advanced resuscitation strategies, and arrhythmia management in previously healthy patients.

## Conclusions

This case demonstrates the life-saving potential of VA-ECMO in managing refractory intraoperative ventricular arrhythmias, highlighting its role as a bridge to recovery and definitive therapies such as ICD implantation. The patient’s rapid recovery of cardiac function emphasizes the possibility of reversible arrhythmia-induced cardiomyopathy, even in previously healthy individuals. Genetic testing and family screening are important, though negative results do not completely exclude inherited arrhythmias. Limitations include the absence of an acute electrophysiological study and delayed ECMO initiation, reflecting real-world logistical challenges. Clinically, the case underscores the importance of vigilant perioperative monitoring, early identification and correction of electrolyte disturbances, and preparedness for advanced mechanical support, informing potential refinements in institutional protocols for high-risk surgical patients.

## References

[1] Holmberg MJ, Ross CE, Fitzmaurice GM (2019). Annual incidence of adult and pediatric in-hospital cardiac arrest in the United States. Circ Cardiovasc Qual Outcomes.

[2] Aziz F, Paulo MS, Dababneh EH, Loney T (2018). Epidemiology of in-hospital cardiac arrest in Abu Dhabi, United Arab Emirates, 2013-2015. Heart Asia.

[3] Schluep M, Gravesteijn BY, Stolker RJ, Endeman H, Hoeks SE (2018). One-year survival after in-hospital cardiac arrest: a systematic review and meta-analysis. Resuscitation.

[4] Hessulf F, Karlsson T, Lundgren P (2018). Factors of importance to 30-day survival after in-hospital cardiac arrest in Sweden - a population-based register study of more than 18,000 cases. Int J Cardiol.

[5] Bhandary SP, Joseph N, Hofmann JP, Saranteas T, Papadimos TJ (2017). Extracorporeal life support for refractory ventricular tachycardia. Ann Transl Med.

[6] Chen CY, Tsai J, Hsu TY, Lai WY, Chen WK, Muo CH, Kao CH (2016). ECMO used in a refractory ventricular tachycardia and ventricular fibrillation patient: a national case-control study. Medicine (Baltimore).

[7] Jolissaint JS, Nehra D (2020). How should a surgeon and anesthesiologist cooperate during intraoperative cardiac arrest?. AMA J Ethics.

[8] (2025). What are the complications of arrhythmia?. https://www.healthline.com/health/arrhythmia/arrhythmia-complications.

[9] Liu G, Xu X, Yi Q, Lv T (2021). The efficacy of catheter ablation versus ICD for prevention of ventricular tachycardia in patients with ischemic heart disease: a systematic review and meta-analysis. J Interv Card Electrophysiol.

[10] Muresan L, Cismaru G, Martins RP (2019). Recommendations for the use of electrophysiological study: update 2018. Hellenic J Cardiol.

[11] Hayward RM, Tseng ZH (2014). Arrhythmias in complex congenital heart disease. Card Electrophysiol Clin.

[12] Ortiz M, Martín A, Arribas F, Coll-Vinent B, Del Arco C, Peinado R, Almendral J (2017). Randomized comparison of intravenous procainamide vs. intravenous amiodarone for the acute treatment of tolerated wide QRS tachycardia: the PROCAMIO study. Eur Heart J.

[13] Sapp JL, Wells GA, Parkash R (2016). Ventricular tachycardia ablation versus escalation of antiarrhythmic drugs. N Engl J Med.

[14] Sun T, Guy A, Sidhu A (2018). Veno-arterial extracorporeal membrane oxygenation (VA-ECMO) for emergency cardiac support. J Crit Care.

[15] Smith M, Vukomanovic A, Brodie D, Thiagarajan R, Rycus P, Buscher H (2017). Duration of veno-arterial extracorporeal life support (VA ECMO) and outcome: an analysis of the Extracorporeal Life Support Organization (ELSO) Registry. Crit Care.

